# A Portable Smart Fitness Suite for Real-Time Exercise Monitoring and Posture Correction

**DOI:** 10.3390/s21196692

**Published:** 2021-10-08

**Authors:** Abdul Hannan, Muhammad Zohaib Shafiq, Faisal Hussain, Ivan Miguel Pires

**Affiliations:** 1 Knowledge Unit of System and Technology, University of Management and Technology, Sialkot 51310, Pakistan; 2 Department of Computer Science and Engineering, Università di Bologna, 40126 Bologna, Italy; muhammadzohaibshafiq@hotmail.com; 3 Al-Khwarizmi Institute of Computer Science (KICS), University of Engineering & Technology (UET), Lahore 54890, Pakistan; 4 Instituto de Telecomunicações, Universidade da Beira Interior, 6200-001 Covilhã, Portugal; 5 Escola de Ciências e Tecnologias, University of Trás-os-Montes e Alto Douro, Quinta de Prados, 5001-801 Vila Real, Portugal

**Keywords:** smart fitness suite, Internet of Things (IoT), human activity recognition (HAR), machine learning (ML), cloud computing, smart gym, posture correction, smart health

## Abstract

Fitness and sport have drawn significant attention in wearable and persuasive computing. Physical activities are worthwhile for health, well-being, improved fitness levels, lower mental pressure and tension levels. Nonetheless, during high-power and commanding workouts, there is a high likelihood that physical fitness is seriously influenced. Jarring motions and improper posture during workouts can lead to temporary or permanent disability. With the advent of technological advances, activity acknowledgment dependent on wearable sensors has pulled in countless studies. Still, a fully portable smart fitness suite is not industrialized, which is the central need of today’s time, especially in the Covid-19 pandemic. Considering the effectiveness of this issue, we proposed a fully portable smart fitness suite for the household to carry on their routine exercises without any physical gym trainer and gym environment. The proposed system considers two exercises, i.e., T-bar and bicep curl with the assistance of the virtual real-time android application, acting as a gym trainer overall. The proposed fitness suite is embedded with a gyroscope and EMG sensory modules for performing the above two exercises. It provided alerts on unhealthy, wrong posture movements over an android app and is guided to the best possible posture based on sensor values. The KNN classification model is used for prediction and guidance for the user while performing a particular exercise with the help of an android application-based virtual gym trainer through a text-to-speech module. The proposed system attained 89% accuracy, which is quite effective with portability and a virtually assisted gym trainer feature.

## 1. Introduction

Human activities recognition (HAR) has gained an immense amount of interest in research due to its tremendous applications in various daily life use cases [1,2,3,4], e.g., human–computer interface, health monitoring, clinical assessments, sports, among others. At different stages of the coronavirus age, outdoor physical activities have been badly affected as parks, gyms, sports clubs, among others, have been closed, and people have had to be restricted to their homes [5,6]. Furthermore, going to a gym or sports club and hiring a trainer is not feasible for everyone due to high membership costs and restricted gym or sports club timings [7]. Thus, many people prefer to exercise at home or in parks or gyms without hiring a training coach [7].

Sports and exercise are very worthwhile for maintaining human health and fitness [8,9]. However, it can be frivolous or even hazardous if the activities are not performed appropriately [5,7]. For example, the weights used in exercise can cause severe muscular issues if a person does not maintain proper posture during intense workouts [7].

**Figure 1:**
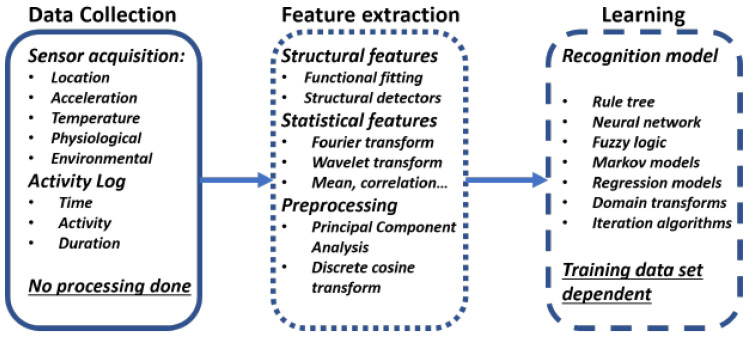
A generic architecture of HAR systems.

Doing exercise at home without a trainer can lead to injuries if someone does it in the wrong manner. However, due to the advancements in sensors technology, it is now possible to monitor human activities through smartphones, wearable devices, environment sensors such as cameras, pressure sensors, ultrasonic sensors, among others [10]. Figure 1 illustrates a generic architecture of the HAR systems. Based on the device deployment and data collection method, the HAR systems are broadly classified into two types, i.e., wearable HAR systems and non-wearable or ambient HAR systems [3,11]. In non-wearable or ambient HAR systems, the sensors, such as cameras, pressure sensors, radar sensors, ultrasonic sensors, among others, are deployed in the environment to monitor human activities [11]. However, the ambient HAR systems are very costly and have limited area coverage that depends upon the underlying sensors used in the system [12]. On the other hand, the wearable HAR systems usually include inertial sensors like accelerometers, gyroscopes, magnetometers, among others, for human activity monitoring. Therefore, the wearable HAR systems are low-cost and do not have any coverage area limit, unlike the ambient HAR systems [12,13].

With the advancement in wearable sensors and ubiquitous computing, sport and fitness have become a notable subject in both research and development [10]. As a result, numerous customer items incorporate hardware with inertial measurement unit (IMU) sensors for fitness tracking and pulse perusing [14]. Furthermore, programming packs empower individuals to relate their movement and required information with their training progresses [15]. For example, an immense range of consumers utilize running trackers with a pedometer (IMU) and GPS-area following. A few weightlifters additionally utilize a smartphone-based training log to monitor their workouts. In any case, the more significant part of the information comes from wearable sensors and client input. The data that can be inserted from them are fundamentally redundancies of a particular movement.

Moreover, physical activities are favorable for one’s well-being and emotional prosperity [16], and are related to improved health conditions, lower mental pressure and tension levels. However, the recent inactive way of life has resulted in obesity, stoutness, type 2 diabetes mellitus, and cardiovascular disease among the vast majority of the inhabitants on the planet, and this has made physical exercise a need rather than an option. Moreover, the World Health Organization’s (WHO) suggestion of a minimum of 150 min high-force physical exercise every day for fit and healthy living has driven individuals to invest both energy and time in physical activity [1,10,17]. Inferable from the propelling consciousness of different advantages of daily workouts, individuals around the globe have relocated to workout centers and body fitness clubs to carry out their regular exercise sessions.

However, while performing high-power and vigorous workouts, there is a high likelihood that physical health is seriously affected. For example, jarring motions and improper posture during workouts can lead to temporary or permanent disability. As a result of technological advances, activity acknowledgment dependent on wearable sensors has attracted many researchers. This undertaking comprises doling out a classification of action to the data given by wearable sensors, such as accelerometer, gyroscope, and EMG.

In the current era, everyone is fond of fitness, wanting to look healthy and smart. Exercising is a way to keep one in shape to be physically and internally fit [8]. Many diseases are cured through exercise, including high blood pressure, diabetes, asthma, and arthritis. However, going to the gym and starting to exercise is not the ultimate fix to the problem. It may lead to even severe cases if not done correctly or under professional supervision. The backbone problem is the most common due to misguidance, overtraining, and bad posture. High-intensity workouts always have a significant impact on the back, and they must be adequately performed. Our solution tackles this problem by collecting data through wearable sensors, classifying it, and sending it to the smartphone using a microcontroller. This smart and intelligent suite acts as a real-life personal trainer by guiding the user and generating alerts in real time.

**Figure 2:**
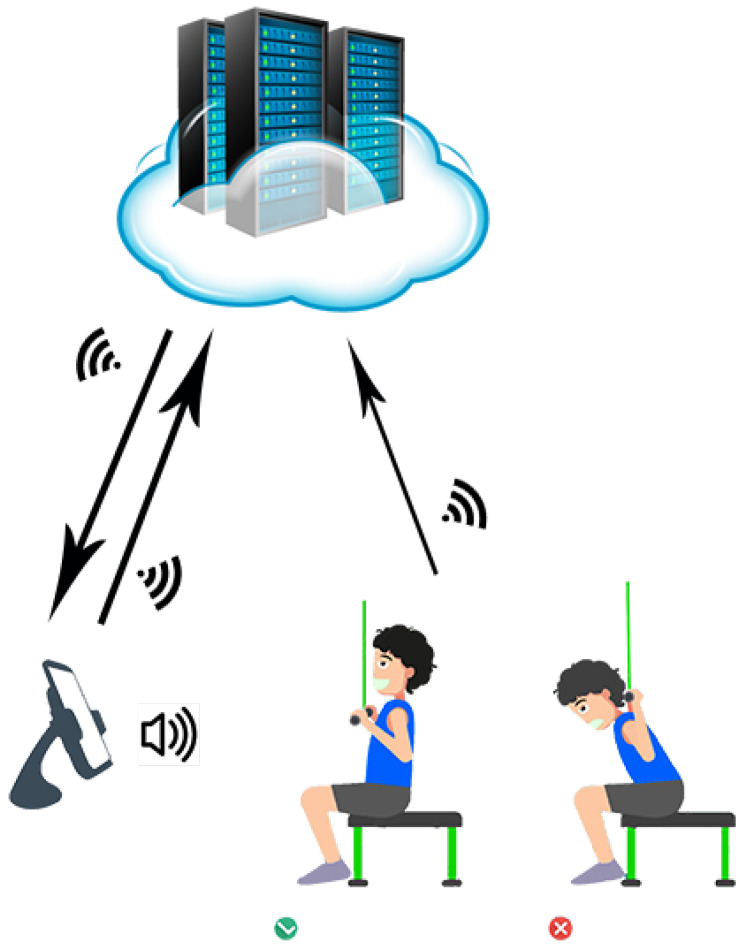
Visualization of proposed system.

Figure 2 envisions the key concept of the proposed system for smart gym exercise monitoring. First, it alerts unhealthy, wrong posture movements over an android application and guides to the best possible posture based on sensor values. Next, the sensors send raw data to the microcontroller, which is further classified and trained. Then, with the help of cloud services, the android application is connected to the sensors and guides the users according to the particular exercise selection.

This research introduces an intelligent suite dependent on IoT innovative technology to give recommendations to an individual during exercise sessions. We seek to aid people in performing the correct posture for exercises by building the Smart Fitness Suite. The software application detects the user’s exercise pose and provides helpful feedback on the user’s stance based on sensor values. The intelligence of the presented system lies with its ability to analyze real-time posture conditions during workouts and predict the most accurate angles. For classification purposes, the proposed framework uses K Nearest Neighbor (KNN) model, Random forest, Deep Neural Networks (DNN), and Linear Regression. The key contributions of the proposed system for gym exercise monitoring are as follows:

- We designed a portable smart fitness suite for sensory data collection while performing two common exercises, i.e., T-bar and bicep curl, respectively.
- To the best of our knowledge, it is first that the posture correction mechanism is proposed through an android-based virtual trainer module.
- Further, we analyzed the performance of the four commonly used machine learning algorithms for classifying the gym exercises.

## 2. Related Work

Smart system applications work phenomenally in every walk of life. Out of them, intelligent healthcare systems are the core interest of researchers nowadays. Due to their sensitivity and effectiveness, smart healthcare frameworks offer effective therapeutic types of assistance in diverse fitness applications. This section reviews the contributions of different analysts in the field of IoT-based smart fitness and human healthcare systems.

Authors in [13,18] worked on recognizing human daily living activities through accelerometer, magnetometer, and gyroscope sensor data. The authors applied different data pre-processing techniques, i.e., normalization and imputation techniques, to efficiently detect human daily living activities. Similarly, authors in [11,12] worked on elderly fall detection through wearable inertial sensors, i.e., accelerometer and gyroscope data. Their proposed solution timely and efficiently detects the elderly fall and generates an alarm to the caretakers of elderly persons to timely rescue the elderly and avoid severe injuries.

Paul Lukowicz et al. [19] exhibited a wearable system based on a textile pressure sensor, which can measure the activity of muscle during different exercises. Thus, it can be used to monitor muscle engagement and check the quality of a workout. Furthermore, the authors proposed a system that keeps track of muscle activity, based on which it can intelligently distinguish between exercises using a pressure mapping sensor. However, a significant loophole in this research work is to detect muscle activity using a pressure sensor instead of electromyography (EMG) sensor, which is state-of-the-art for measuring muscle activity. Unfortunately, we cannot achieve perfection through a textile mapping sensor since it measures pressure distribution on the human body instead of electrical signals generated by muscles.

Similarly, in [20], the author proposed a real-time smart gym application that obtains the values of sensors from a smart fitness watch during a workout. It further converts the time-critical values into waveform to display them on application. The major drawback of this application is that it adopted a time trigger mechanism only, which might be deadly during a workout. Moreover, as time progresses, the wrong posture hurts the body. In addition, it provided exercise training under the supervision of GYM trainer during exercise sets. So, to incorporate this issue, our proposed system adopted a hybrid approach that includes both time and event trigger information. We import the pre-trained model inside the application to eliminate gym experts during workouts and make our system more user-friendly, efficient, and effective.

The authors in [9] worked on recognizing gymnastic actions through an artificial neural network (ANN). The authors proposed ANN-based hardware for fast and accurate gymnastic action detection, i.e., field-programmable gate array (FPGA). Likewise, the authors in [21] presented a health powerlessness expectation model that inspects ongoing health during the training period by using IoT architecture. The recommended model analyses diverse health-centered credits to determine the health state defencelessness proficiently. Moreover, the model’s architecture is made to perform distinct predefined undertakings synchronized to achieve general adequacy. At first, IoT technology is used to gather data for various traits during exercise. Then, these data are transferred to the cloud for inside-out examination. However, portability and robustness were not considered in this research work.

Likewise, the authors in [10] proposed a methodology to detect and recognize four types of workout activity through a single three-channel accelerometer worn on the chest. The authors used a deep convolutional neural network (DNN) and achieved 89.9% accuracy for recognizing four types of activity. Likewise, the authors of [7] proposed a wearable device called fitness coach, which identified 12 different kinds of exercise. The proposed system is capable of providing review scores after assessing the performed activity. Further, it gives some recommendations for the user to complete the practice in a better way.

The authors in [22] focused on detecting the biceps muscle fatigue while performing bicep-related exercises at the gym. The authors extracted 33 features and selected 16 useful features to train and test five different machine learning (ML) models to detect bicep muscle fatigue. Among these, two-layer feed-forward neural networks outperformed all other ML models with 98% accuracy. Similarly, authors developed a methodology to efficiently detect four different functional fitness exercises using inertial sensors [1]. Their proposed method achieved 96.4% accuracy with one-sensor data and 97.6% accuracy with two-sensor data using an SVM classifier.

In addition, [8,23] introduced Pose Trainer. This end-to-end computer vision application uses pose estimation, visual geometry, and machine learning to provide personalized feedback on fitness exercise form. They used the output of pose estimation to evaluate videos of exercises through human pose key points. They worked with four different exercises, recording training videos for each. Next, they used geometric heuristic algorithms to provide personalized feedback on specific exercise improvements and machine learning algorithms to determine posture correctness using only labeled input videos automatically.

Sadeka Haque et al. [24] proposed a deep neural network (DNN)-based mechanism for exercise posture analysis that speaks to an approach to distinguish human posture from 2D human activities. The frameworks based on deep learning are making it practical to recognize exercise presents from pictures. They assessed their proposed model on the dataset that contains an aggregate of 2000 pictures. Moreover, those pictures are scattered into five classes just as images are assigned to prepare and test datasets and improve execution. They have directed different examinations with their model on the validation dataset, obtaining precision of 82.68%. However, their proposed model could not detect some poses from images due to over-fitting. The user would have to save pictures to the data-set each time while training, which is irritating and time-consuming. The suggested model is perplexed to recognize some poses due to the over-fitting issue.

Based on the limitations of existing work, our fitness suite is embedded with multiple accelerometers, gyroscopes, and EMG sensors to send real-time body posture data on user applications. Its other process on the cloud platform to generate fruitful recommendations about correct body posture. As a result, our proposed fitness suite surpasses the existing smart gym systems in latency, energy efficiency, cost-effectiveness, and portability.

## 3. Proposed Methodology

**Figure 3:**
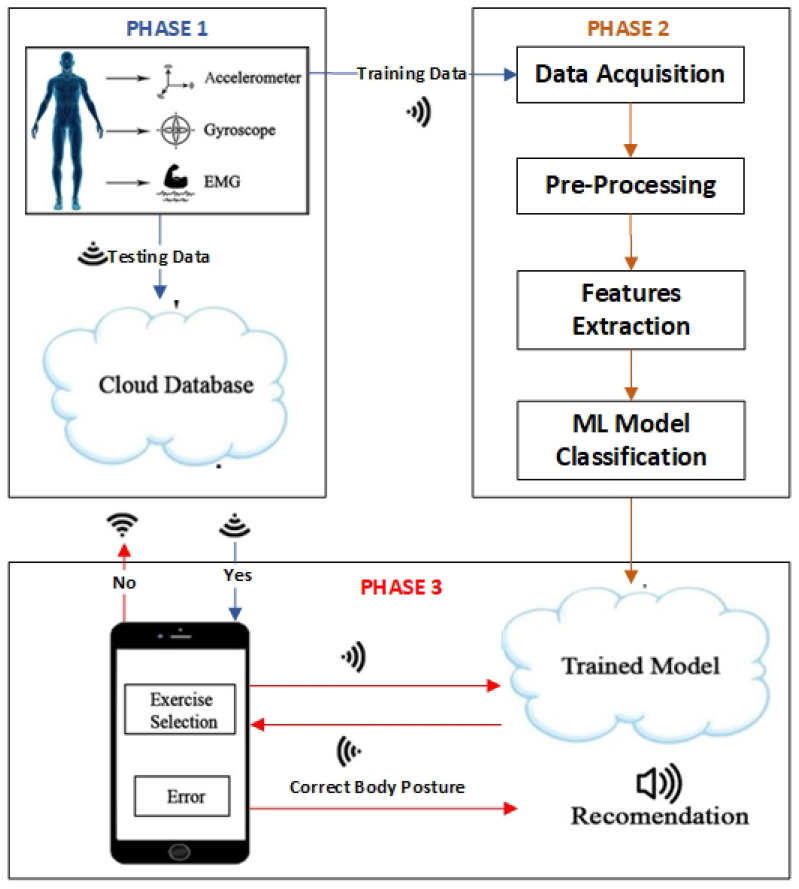
Proposed methodology for smart fitness recommender system.

The proposed smart fitness suite for correct body posture recommender system is divided into three major phases, i.e., Smart Fitness Suite Clothing, Data Processing and Classification, and Real-Time Exercise Monitoring and Posture Correction, as shown in Figure 3.

### 3.1. Phase 1: Smart Fitness Suite Clothing

**Figure 4:**
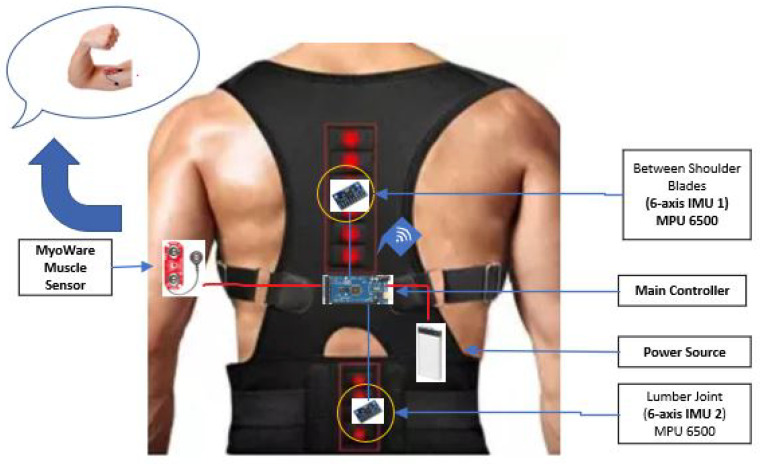
Sensor orientation on smart fitness suite.

**Figure 5:**
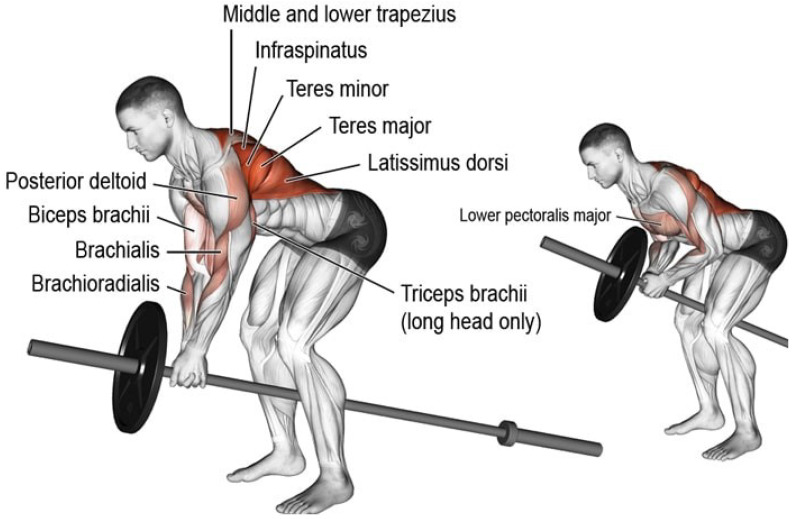
Muscles used in T-bar exercise.

**Figure 6:**
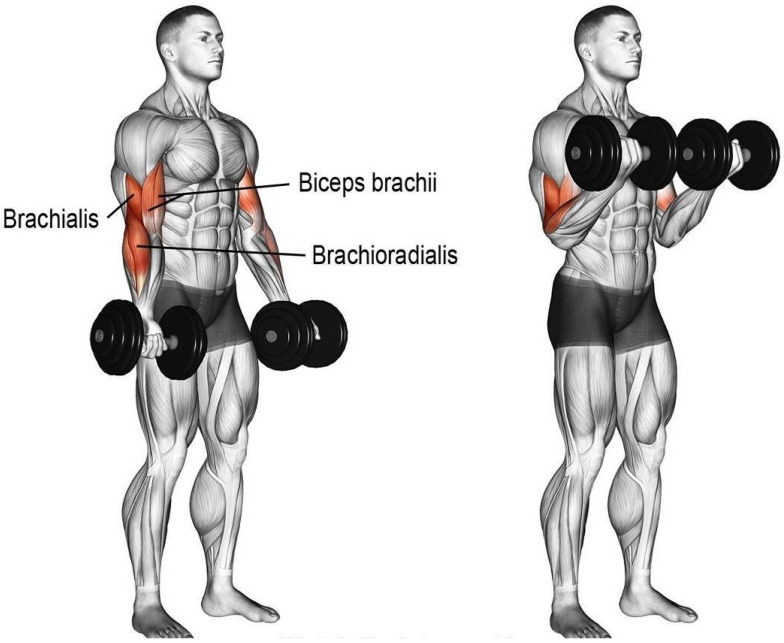
Muscles used in bicep dumbbell curl exercise.

Wearing the smart fitness suite is a primary phase of the proposed methodology to proceed with subsequent steps. The proposed smart fitness suite is embedded with two inertial measurement unit (IMU) sensors (i.e., MPU 6500) and an electromyography (EMG) sensor. Each IMU sensor consists of one accelerometer sensor and one gyroscope sensor. Based on the existing research works [3,25,26], we used only 3-axis gyroscope signals of each IMU sensor for monitoring the movement of teres and latissimus muscles while the EMG signals to monitor the movement of bicep muscles. The two gyroscope sensors are located between the upper back shoulder blades and the middle back lumbar support of the smart fitness suite, as displayed in Figure 4. The upper gyroscope sensor, i.e., IMU-1, monitors the movement of teres major muscle and teres minor muscle, as shown in Figure 5. On the other hand, the lower gyroscope sensor, i.e., IMU-2, monitors the movement of the latissimus dosri muscle. Similarly, the EMG sensor monitors the movement of biceps brachii muscles, as shown in Figure 6.

We trained our fitness suite for two exercises, i.e., T-bar and bicep concentrated dumbbell curl. The primary muscles involved in T-Bar exercise are latissimus dosri, teres major, and teres minor, shown in Figure 5. However, for the bicep dumbbell curl exercise, we used an EMG sensor (MyoWare) placed on the bicep brachii muscles to monitor the electromyography of contraction and relaxation of the bicep muscles, shown in Figure 6.

The data recorded through the smart fitness suite are transmitted to phase 2 and cloud database for training and testing purposes. In phase 2, data are acquired in the data acquisition module, which is used for data training under the supervision of a certified gym instructor. On the other hand, test data are stored on the cloud database, further fetched in phase 3. The whole process takes approximately 700 ms over a 12 MB bandwidth internet connection.

### 3.2. Phase 2: Data Processing and Classification

The whole machine learning (ML) classification process is executed in this phase, subdivided into four stages, i.e., Data Acquisition, Pre-processing, Features Extraction, and ML model Classification.

#### 3.2.1. Data Acquisition

In this stage, sensory data of two gyroscope sensors, i.e., IMU-1 and IMU-2, are acquired through a smart fitness suite for T-bar exercise. Furthermore, EMG data of bicep brachii muscle through the MyoWare muscle sensor module are acquired to train the bicep dumbbell exercise. The data were collected from 9 participants aged 20–30 years who performed these exercises under the supervision of two professional gym trainers. Each participant wore the proposed fitness suite and performed three sets of each exercise. After performing one set of an exercise, the user relaxed for 60 s and then started performing the next set. In addition, the T-bar and bicep dumbbell curl exercise sensory data-set was collected from free weights up to 10 kg during exercise sets.

Moreover, each participant had 1–2 years of experience in gym training. The complete dataset is gathered on the IoT analytic platform, i.e., ThingSpeak. This dataset is used to train the particular exercise data for the ML classification mechanism.

#### 3.2.2. Pre-Processing

In this stage, the acquired sensory data are pre-processed to refine the data for better classification. For this purpose, we first removed the NULL values. Afterward, we removed the redundant values. Finally, we cleaned the dataset by removing the malformed values.

#### 3.2.3. Feature Extraction

In this stage, we extracted seven features from the embedded sensory module, which are calculated based on the angular rotation about X (pitch), Y (yaw), and Z (roll) plane values of two gyroscope sensors and one EMG sensor, i.e., MyoWare muscle sensor. These features include delta x, i.e., the difference in angular rotation of two gyroscope sensors around X plane, delta y, i.e., the difference in angular rotation of two gyroscope sensors around y plane, and delta z, i.e., the difference in angular rotation of two gyroscope sensors around the Z plane, relative radial distance (r), i.e., the relative radius of each angular plane of two gyroscope sensors, the angular distance (θ), i.e., the angular distance in three axes (i.e., XYZ plane) of two gyroscope sensors, mean absolute value (MAV), i.e., an average of the absolute value of EMG signal, and root mean square (RMS), i.e., square root of the mean square of EMG signal. These features are described in mathematical expression in Table 1.

#### 3.2.4. ML Model Classification

The complete dataset is classified on the particular ML-based classification model. For this purpose, we selected four commonly used classifiers, i.e., logistic regression (LR), Support vector machine (SVM), K-nearest neighbor (KNN), and Random Forest classifier (RFC). Then, we split our dataset into 80:20 ratio, i.e., 80% data used for training and 20% data for testing. Next, we trained and tested all four previously mentioned classifiers on the collected data. The training and testing are performed over the cloud server. Finally, we selected the best classifier and embedded it in an Android application for real-time exercise recommendation.

### 3.3. Phase 3: Real-Time Exercise Monitoring and Posture Correction

Eventually, as previously discussed, the best ML classifier resulting from phase 2 is imported into the Android application. Then, the Android application takes the real-time data of the user while performing the exercise. Gathering data from the two inertial sensors, one EMG sensor, and sending it to the cloud storage until the prediction of a user’s exercise takes 700 ms as discussed in Section 3.1. Finally, the recommended output about correct body posture is conveyed to the user through the text-to-speech feature of a user application. It acts as a virtual gym trainer that properly guides the user throughout the exercise training session.

### 3.4. Workflow of System

**Figure 7:**
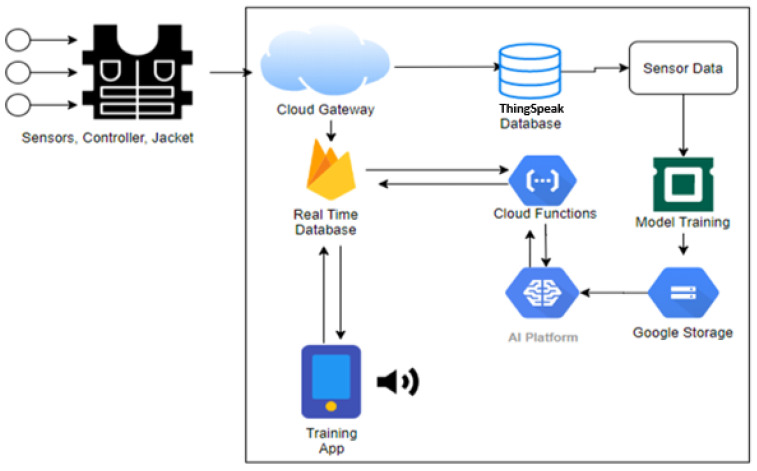
Proposed smart fitness architecture flow diagram.

Figure 7 represents the overall flow of the proposed system. The proposed approach comprises of two main phases, i.e., dataset gathering for model training and the portable smart fitness suite prototype for user testing. In the first phase, gym exercises, i.e., T-bar and bicep sensory data, are gathered through a ThingSpeak cloud analytic platform. Then, after proper data cleaning of the acquired sensory dataset in its pre-processing stage, the dataset is trained on the ML-based classification model through the Google Cloud AI platform. Moreover, the predicted results received from the AI platform to send to the Android application. Finally, the proposed model is tested through obtained predictions on dedicated user android application account through a training process and assist the user through text speech feature on an android application in real-time.

### 3.5. Proposed Algorithm

Algorithm 1 represents the pseudo-code of the proposed methodology for workouts body posture recommendation system. Initially, the sensors embedded in the fitness suite are used to attained real-time data. Afterward, if the sensor’s value is greater than the initial value, i.e., “0”, the sensory data is passed to the real-time database for storage and fetching. Finally, the user android application received the input data from the real-time data storage and handed it to the AI module of the cloud server. Then, the best ML classifier is trained on the sensors data, i.e., KNN in our case, to guides the user based on predictions while performing an exercise. We used three predictions actions, i.e., 2, 3, and 4. Based on them, a complete user guidance mechanism was developed.

Moreover, a particular action is performed on each prediction, i.e., prediction equals 2 represents the perfect position of the selected exercise, with no need to change the body posture. Furthermore, prediction equals 3, guides the user to straighten their lower back and chest up to attain the exact position in T-bar exercise or to rest in the case of bicep dumbbell curl exercise. Finally, prediction equals 4, which represents needing to bend more to be in a perfect posture for the T-bar exercise. The user’s Android application is integrated with the AI module of the cloud server and real-time database, which act as a bridge between a cloud AI platform and an Android application. Finally, the text-to-speech feature makes recommendations and guides the user based on the trained classification model concerning a selected exercise in real time.
**Algorithm 1** Pseudo code of the Proposed System**Input:** Gyroscope Sensor and Electromyography sensor**Output:** Recommendation on text view, Recommendation by text-to-speech feature 1: Initialize sensor value equals to zero 2: **if**
 Checksensorvalue>0 
**then** 3:  Input passed to Real-Time Database 4: **end if** 5: **repeat** 6:  Android receive input from Real-Time Database 7:  Data passed to AI Platform through cloud functions 8:  Classification Algorithm = KNN 9:  Prediction forwarded to Android through cloud functions 10:  **if**
 prediction==2 
**then** 11:   Perfect || Keep going 12:  **else if**
 prediction==3 
**then** 13:   Please straighten your lower back and chest up || You must rest 14:  **else if**
 prediction==4 
**then** 15:   Please bend a little 16:  **else** 17:   Junk values 18:  **end if** 19: **until** exercise  ||  (timer>0) 

## 4. Experimental Setup

In the proposed fitness suite, we aim not only to suggest correct posture but also to detect mistakes and guide the user accordingly. For the attainment of data, our system used only two 3-axis gyroscope signals of each IMU, i.e., IMU-1 and IMU-2, and one EMG MyoWare muscle sensor module. The two 3-axis gyroscope signals of each IMU sensor are based on orientation concerning gravity for posture detection, which is helpful for T-bar exercise monitoring, as discussed in Section 3.1 Furthermore, the proposed smart fitness suit is embedded with a Wi-Fi module (i.e., NodeMCU Esp 8266 v3) to send data to the IoT analytic platform (i.e., ThingSpeak). The two 3-axis gyroscope sensors and an EMG sensor are placed in the appropriate position after consultation with a certified fitness trainer, as highlighted in Figure 4.

**Figure 8:**
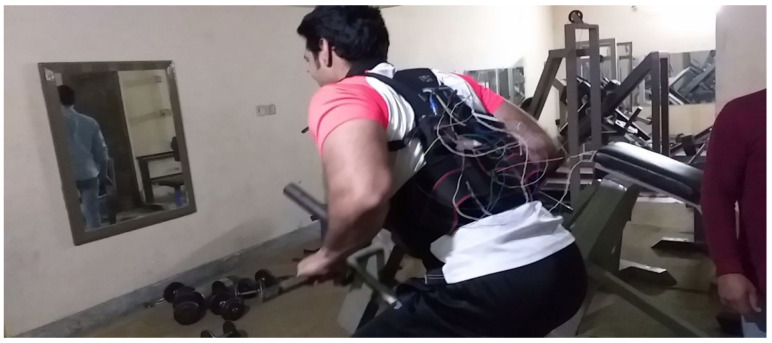
Experimental setup.

All these sensors are embedded in the fitness suite attached with the central control unit (Arduino Mega 2560) worn by 9 volunteers to performed exercises, based on which the whole dataset is attained. This dataset is used to train on the best classification model on an AI module of Google Cloud Platform (GCP), as discussed in Section 3.2.4. Moreover, these participants performed standing T-bar, and bicep concentrated dumbbell curl exercise for several days, two hours each day, as shown in Figure 8. Afterward, three classes were made for this dataset to guides the user, i.e., one for perfect posture and the other two representing common mistakes while performing user exercise as mentioned in Section 3.5. Finally, the guided output is sent to the user Android application through text-to-speech feature in real time.

### 4.1. Performance Metrics

Statistical validation of our collected dataset and evaluation parameters used for proposed smart fitness architecture are as follows:

#### 4.1.1. *T*-Test

This test is used to determine the validity of the collected smart gym dataset based on the null hypothesis. *T*-test gives us the probability of statistical difference based on which we determine the behavior of different classes of data that is either that they are biased on unbiased. Mathematically it is represented by Equations (1)–(4).

(1) $$t=\frac{\left(\bar{x1}-\bar{x2}\right)-\left(\mu 1-\mu 2\right)}{s\sqrt{\frac{1}{n1}+\frac{1}{n2}}}$$

(2) df=n1+n2−2

(3) $$s^{2}=\frac{\left(n1-1\right)\times s1^{2}+\left(n2-1\right)\times s2^{2}}{\left(n1-1)+(n2-1\right)}\;\mathrm{where}\;\;s=\sqrt{s^{2}}$$

(4) α=0.05

where $\bar{x1}$ and $\bar{x2}$ are the mean values of two datasets in our case, i.e., two classes, whereas n1 and n2 are the numbers of samples. Moreover, μ1−μ2 is the hypothesis that we assumed to be null, i.e., (μ1−μ2=0) and df is the degree of freedom that determines how much data could vary inside a given sample. We compare the value of df and *t* from [27] to find the value of *p*, which represents its corresponding probability. Finally, if the *p*-value is >α, we fail to reject the hypothesis, and if the *p*-value is <= α, we reject the null hypothesis with at least 95% confidence that our data is valid and unbiased.

#### 4.1.2. Precision (P)

The precision parameter is the ratio between actual positives among all which that classifier has predicted as positive. Mathematically it is represented by Equation (5).

(5) $$\begin{array}{c} P=\frac{TruePositive}{TruePositive+FalsePositive} \end{array}$$

#### 4.1.3. Recall (R)

Recall parameter is the ratio b/w true positive results that the classifier has predicted from actual positives. Mathematical representation is shown in Equation (6).

(6) $$\begin{array}{c} R=\frac{TruePositive}{TruePositive+FalseNegative} \end{array}$$

#### 4.1.4. F1-Score (F)

We often have low precision and high recall or inversely high precision and low recall. In this case, it is difficult to determine the validity of a model. Hence, we use the F1-score to assess its performance. F1-score is the combined harmonic mean of precision and recall performance metrics. However, high precision and high recall result in a high F1-score. Therefore, we could quickly evaluate our model based on the F1-score. Mathematical representation is shown in Equation (7).

(7) $$\begin{array}{c} F=\frac{2*P*R}{P+R} \end{array}$$

#### 4.1.5. Accuracy (A)

Accuracy is the ratio of true positive results out of the complete smart gym sensory dataset. Mathematical representation is shown in Equation (8).

(8) $$\begin{array}{c} A=\frac{TruePositive+TrueNegative}{TruePositive+TrueNegative+FalsePositive+FalseNegative} \end{array}$$

## 5. Performance Analysis

The proposed smart and portable gym mechanism for user assistance is evaluated based on four performance metrics as mentioned in Section 4.1 and the complete dataset is statically validated through the *t*-test procedure. The expressive representation of each parameter is shown below.

### 5.1. Statistical Dataset Validation (T-Test Procedure)

**Figure 9:**
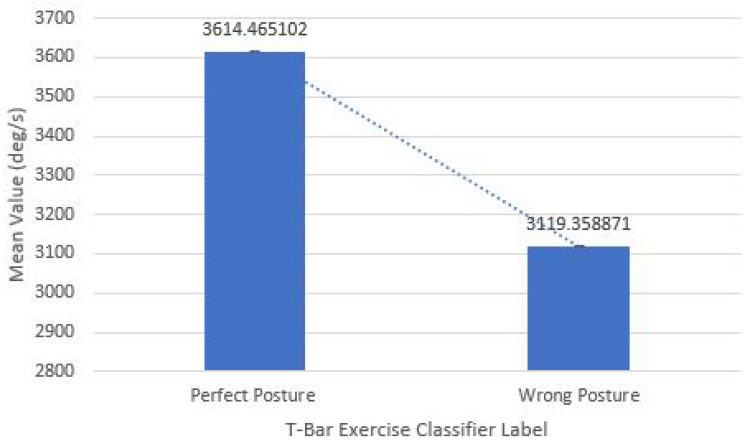
Difference in mean values of gyroscope based sensory dataset classes.

**Figure 10:**
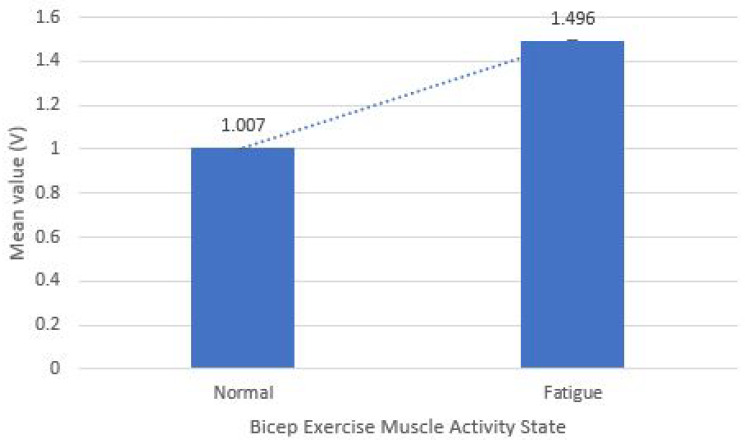
Difference in mean value of EMG based sensory dataset classes.

In this section, the sensory gym dataset of gyroscope and EMG modules is validated statistically by a two-tailed *t*-test for critical distribution between different data classes. Table 2 displays the results of the test (*p*-value), which proved that the chance of error in collecting different states of data is 0.02 for gyroscope sensor and 0.0001 for EMG sensor both are lesser than 0.05, which is a standard scale for rejecting null hypothesis, thus, we could say that data collected for perfect and wrong body posture, along with different states of muscle, have a meaningful variation with more than 97% confidence. Furthermore, apart from the previous test, the significant difference represented by a dotted line in the mean values of two states of muscle and body posture also proves that data is unbiased and statistically valid as shown in Figure 9 and Figure 10.

### 5.2. Percentage System Accuracy (PSA)

**Figure 11:**
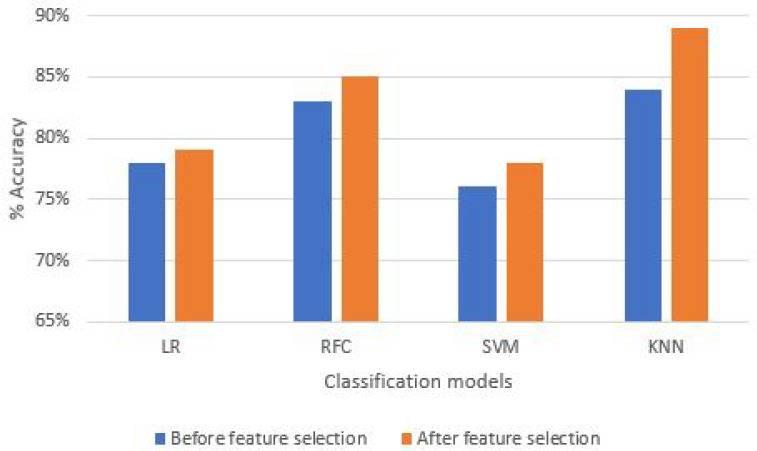
Percentage system accuracy of T-bar exercise before and after feature selection.

We evaluated the accuracy of the proposed system on four major ML classification models, as shown in Figure 11. In the first step, data are classified without any filtration technique having the same number of attributes. Afterward, the dataset is split according to the 80:20 rule, where 80% of data are used for training, and the other 20% of data are used for testing as discussed in Section 3.2.4. Finally, we classified the proposed fitness model based on K-nearest neighbor (KNN), logistic regression (LR), random forest classifier (RFC), and support vector machine (SVM) ML classification methods. The visual representation of these ML classification models is shown in Figure 11.

Moreover, 10 trees are used for RFC during classification, resulting in the highest accuracy. On the other hand, in KNN, three nearest neighbors are specified, resulting in the highest accuracy. The forward feature selection technique selects the most influencing features in the dataset, resulting in increased accuracy for the training model. Subsequently, adding feature one at a time to the dataset while training, we eliminated the Y-axis gyroscope values for the upper back in the T-bar exercise. Considering this feature did not result in any significant increase in accuracy parameter. Figure 11 shows the results of different classification models based on the before and after feature selection mechanism highlighted with blue and orange bars. It is analyzed that KNN resulted in the highest individual accuracy of 89%. In contrast, the RFC is slightly lower than KNN with 85% accuracy while the other two classifiers, i.e., LR, and SVM have significantly lower accuracy values, i.e., 78 % and 77%, respectively. To conclude, after feature selection, the PSA of the KNN ML classification model increases significantly, resulting in the best suitable ML model selected for the proposed fitness architecture. However, in the case of the bicep dumbbell curl exercise, the maximum accuracy achieved from KNN, i.e., 98%, as compared to all other ML classification models as shown in Table 3.

### 5.3. Percentage System Precision (PSP)

**Figure 12:**
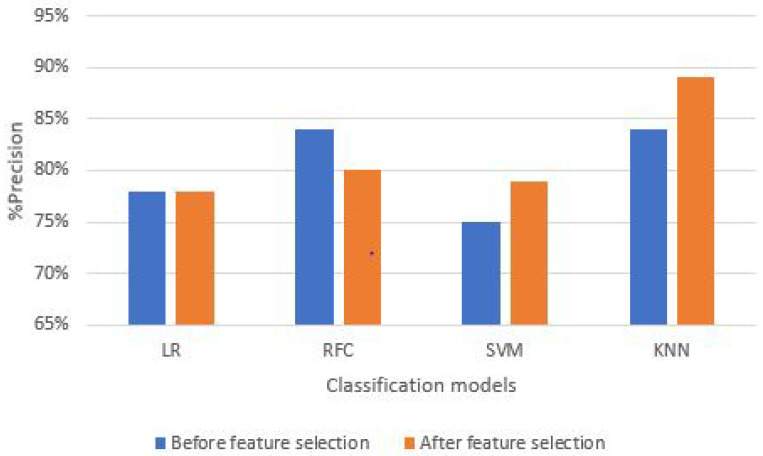
Precision of classification models before and after feature selection.

Likewise, PSA, for percentage system precision parameter (PSP), the KNN classification model again proved the best fit for the proposed fitness suite for both T-bar and bicep gym exercises. Figure 12 and Table 3 represent the comparison between commonly used ML classification models for both exercises. We achieved the highest percentage of precision while adopting the after feature technique from the KNN model, i.e., 89% for the T-bar exercise, and with before feature selection, we achieved 84%. In addition, in the bicep dumbbell curl exercise scenario, we achieved 98% precision from the KNN model, which surpasses all other ML classification models.

### 5.4. Percentage System Recall (PSR)

**Figure 13:**
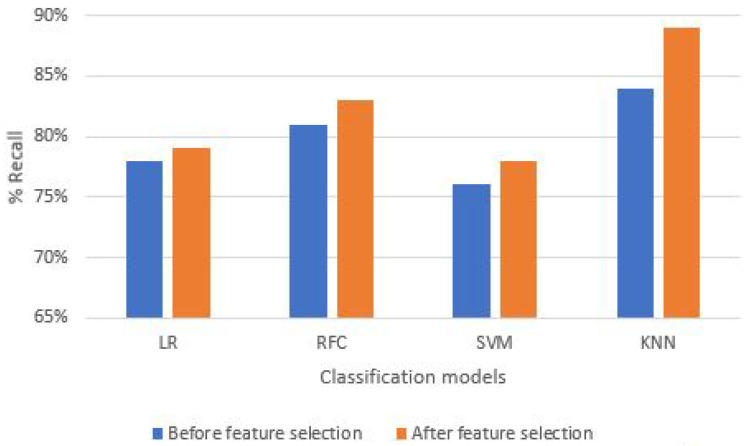
Rate of recall of classification models before and after feature selection.

In relation to the above ML classification models response, KNN again proved to be the best-fit classifier in the case of PSR for both T-bar and bicep exercises, as shown in Figure 13 and Table 3. The highest percentage recall in both before and after feature selection mechanism, the T-bar exercise reached 84% and 89%, respectively. However, in the case of the bicep dumbbell curl exercise, we reported the highest achieved PSR equal to 98%, which is quite fascinating.

### 5.5. Percentage F1 Measure (PFM)

**Figure 14:**
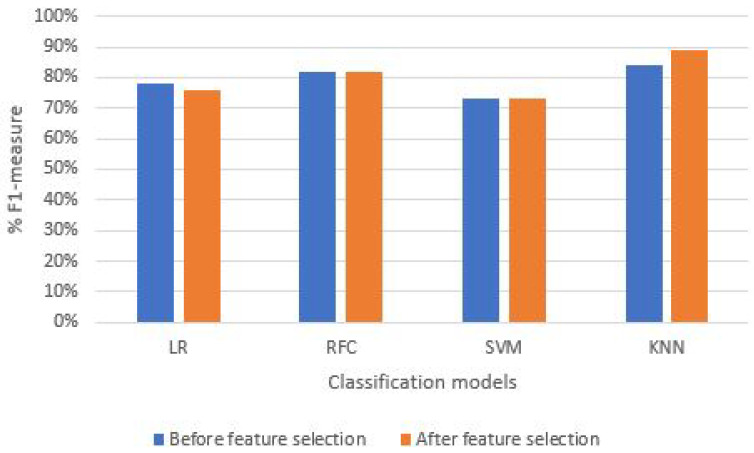
Rate of F1-measure before and after feature selection.

The F1-measure performance parameter is the harmonic mean of PSR and PSP, shown in Section 4.1.4. As illustrated in Figure 14 and Table 3, the KNN ML classification model proved to be the best fit for both exercises mentioned above. In the case of the T-bar exercise, we achieved the maximum PFM from the KNN model is 84% and 89%, respectively, for both before and after feature selection techniques. On the other hand, in the case of bicep exercise, the maximum PFM value achieved is 98%.

## 6. Conclusions

This work presents a body posture smart recommendation system, which detects users’ posture and guides them according to the selected back exercise using a gyroscope sensory module embedded in the smart fitness suite. Along with this, a bicep curl muscle health detection feature is added in the proposed system, which detects muscle health in real time. Furthermore, an EMG sensor stops the user from exercising in the extreme fatigue stage to prevent muscle injury. The collected sensory dataset is analyzed and validated statistically using a *t*-test model. Then, the best classification model is selected by comparing performance metrics based on scientifically suggested parameters such as Precision, Recall, F-Measure, and Accuracy. Finally, after a comparative analysis, KNN proved to be the best fit model with the highest performance with 89% accuracy while using the forward feature selection technique. Subsequently, user-guided recommendations are made by the system based on the trained dataset over the android application using the text to speech feature in real-time. Apart from this, the Android application keeps track of user workout reports, analyzes the user’s BMI, and motivates daily workout challenges.

In the future, the smart fitness suite could be trained not only for back or bicep workouts but for other body workouts too. Furthermore, to increase the accuracy of the proposed system, we can consider the gyroscope drift issue to stabilize the signals for better classification of exercises. Finally, the proposed smart fitness suite can be made specifically for male or female users by collecting datasets separately. This would be a massive innovation in health, fitness, and technology.

## Figures and Tables

**Table 1:** Features description of smart fitness suite.

Feature Name	Description	Expression
Delta x	Difference in angular rotation of IMU-1 and IMU-2 around X plane	δx=x2−x1
Delta y	Difference in angular rotation of IMU-1 and IMU-2 around Y plane	δy=y2−y1
Delta z	Difference in angular rotation of IMU-1 and IMU-2 around Z plane	δz=z2−z1
Relative Radial Distance	Relative radius of each angular plane of two gyroscope sensors	$r=\sqrt{{\left(x2-x1\right)}^{2}+{\left(y2-y1\right)}^{2}+{\left(z2-z1\right)}^{2}}$
Angular Distance	Angular distance in three axes (i.e., XYZ plane) of two gyroscope sensors	$\theta =\arccos\left|\frac{x1*x2+y1*y2+z1*z2}{\sqrt{x1^{2}+y1^{2}+z1^{2}}\sqrt{x2^{2}+y2^{2}+z2^{2}}}\right|$
Mean Absolute Value (MAV)	Average of the absolute value of the EMG signal	$MAV=\frac{1}{N}\sum_{n=1}^{N}\left|x_{i}\right|;x=signal,N=samples$
Root Mean Square (RMS)	Square root of the mean square of the EMG signal	$RMS=\sqrt{\frac{1}{N}\sum_{n=1}^{N}x^{2}}$

**Table 2:** *t*-test probability of different dataset classes.

Sensory Dataset Class	*t*-Test Probability Value	Confidence Level
Gyroscope sensor ata	0.02	98%
EMG sensor data	0.0001	99.9%

**Table 3:** Performance metrics comparison for bicep exercise sensory dataset with regards to ML classification models.

Classifier	Precision	Recall	F-Measure	Accuracy
LR	92%	90%	90%	90%
RFC	96%	96%	96%	96%
SVM	95%	94%	94%	94%
KNN	98%	98%	98%	98%
